# Computational modeling of the immune response in multiple sclerosis using epimod framework

**DOI:** 10.1186/s12859-020-03823-9

**Published:** 2020-12-14

**Authors:** Simone Pernice, Laura Follia, Alessandro Maglione, Marzio Pennisi, Francesco Pappalardo, Francesco Novelli, Marinella Clerico, Marco Beccuti, Francesca Cordero, Simona Rolla

**Affiliations:** 1 Department of Computer Science, University of Turin, Turin, Italy; 2 Computer Science Inst., DiSIT, University of Eastern Piedmont, Alessandria, Italy; 3 Department of Clinical and Biological Sciences, University of Turin, Orbassano, Italy; 4 Department of Drug Sciences, University of Catania, Catania, Italy; 5 Department of Molecular Biotechnology and Health Sciences, University of Turin, Turin, Italy

**Keywords:** Multiple sclerosis, Immune system, Computational modeling, Stochastic modeling, Petri net

## Abstract

**Background:**

Multiple Sclerosis (MS) represents nowadays in Europe the leading cause of non-traumatic disabilities in young adults, with more than 700,000 EU cases. Although huge strides have been made over the years, MS etiology remains partially unknown. Furthermore, the presence of various endogenous and exogenous factors can greatly influence the immune response of different individuals, making it difficult to study and understand the disease. This becomes more evident in a personalized-fashion when medical doctors have to choose the best therapy for patient well-being. In this optics, the use of stochastic models, capable of taking into consideration all the fluctuations due to unknown factors and individual variability, is highly advisable.

**Results:**

We propose a new model to study the immune response in relapsing remitting MS (RRMS), the most common form of MS that is characterized by alternate episodes of symptom exacerbation (relapses) with periods of disease stability (remission). In this new model, both the peripheral lymph node/blood vessel and the central nervous system are explicitly represented. The model was created and analysed using *Epimod*, our recently developed general framework for modeling complex biological systems. Then the effectiveness of our model was shown by modeling the complex immunological mechanisms characterizing RRMS during its course and under the DAC administration.

**Conclusions:**

Simulation results have proven the ability of the model to reproduce in silico the immune T cell balance characterizing RRMS course and the DAC effects. Furthermore, they confirmed the importance of a timely intervention on the disease course.

## Background

In the last years computational models have acquired a pivotal role to assure the safety and effectiveness of medical treatments independently from the biological, immunological, epigenetic and environmental features characterizing each patient. Recently, many regulatory agencies are opening to put beside clinical trials the large scale simulations on virtual patients. The in silico models are decisive when the individual variability can not be captured due to the lack of information about the disease, or simply because of unpredictable environmental events; for these reasons some of the individuals variability can only be captured by exploiting stochastic approaches.

Computational models could give a considerable advance in the study of diseases characterized by a partially understood etiology, for example Multiple Sclerosis (MS). MS is a chronic and potentially highly disabling disease with considerable social impacts and economic consequences. Since more than 700,000 European people suffer from MS, it is the leading cause of non-traumatic disabilities in young adults [1].

The scientific community agrees that MS involves a process mediated by immune system in which an abnormal response of the body’s immune defense is directed against the Central Nervous System (CNS) suffer which is made up of brain, spinal cord and optic nerves. Within the CNS the immune system activates an inflammation process that damages the myelin (i.e. the fatty substance that surrounds and insulates the nerve fibers), the nerve fibers themselves and the cells specialized in myelin production (i.e. Oligodentrocytes (ODC)).

The myelin degradation process is mediated by self-reactive T cells which are activated in the peripheral lymph nodes and secrete pro-inflammatory cytokines (mainly Interferon gamma (IFN$\gamma$) and Interleukin-17 (IL-17)). Among them IL-17 producing T cells sustain the pathogenesis of MS by promoting Blood-Brain Barrier (BBB) disruption and inducing autoimmune inflammation in the CNS [2, 3]. Furthermore, IL-17 producing T cells are increased in the peripheral blood [4, 5], in the cerebrospinal fluid and in the CNS perivascular space of MS patients [5, 6]. These self-reactive T cells can be found also in healthy subjects but are strictly controlled by various mechanisms including suppression by T Regulatory (Treg) cells. In MS patients Treg cells are impaired in number and function [7] and allow self-reactive T cells to expand in the periphery, cross the BBB and reach the CNS, where they undergo into a secondary re-activation and induce demyelination and axonal damage [8, 9]. The triggers that convert the innocuous self-reactive T lymphocytes into pathogenic are still not understood, but a combination of genetic and environmental factors (e.g. Epstein-Barr Virus, vitamine D and smoking) [10] seems to be implicated.

The alteration or completely interruption of nervous messages within the CNS produces a variety of neurological symptoms that will vary among MS patients in type and severity. MS patients typically experience one of four disease patterns (types of MS) with a predominance of the Relapsing Remitting Multiple Sclerosis (RRMS) course observed in approximately 85% of patients at diagnosis [11]. RRMS patients alternate episodes of symptom exacerbation (relapses) with periods of disease stability with complete or partial recovery (remissions) [1].

Until now a dozen treatments have been proposed to reduce the frequency of MS relapses, slow the accumulation of disabilities and contrast the RRMS progression. Such disease modifying therapies include oral agents and monoclonal antibodies (mAbs), which have been designed for a selectivity of drug action. Among mAbs Daclizumab (DAC) was selected for its ability to bind the CD25 sub-unit of the high-affinity Interleukin-2 receptor (IL-2R). IL-2R is a receptor-structure able to bind a key component of the immune system, Interleukin-2 (IL-2), a cytokine that allows T cell proliferation. DAC introduced a new mechanism of action preventing the binding of IL-2 to its receptor with a consequent effect on immune cells which involves the blockade of T effector cells activation, the reduction of Treg cells and the increase of a particular Natural Killer (NK) cells subset with regulatory ability [11]. DAC efficacy was demonstrated in reducing (i) the clinical relapse rate of RRMS, (ii) the disability progression, and (iii) in improving health-related quality of life [11]. DAC appears to be generally well tolerated by MS patients with some adverse events as infections, encephalitis, and liver damages. However, the safety and efficacy results obtained after eight years of DAC treatment from the clinical trials were finally published [12]. The study and the simulation of RRMS course during DAC administration involves a series of hypotheses about the disease mechanisms that cannot be always described through a deterministic process. Environmental events, the complex balance mechanism between the T Effector (Teff) and Treg cells, or the random spreads of self-reactive T cells cannot be well predicted, and fall within that part of uncertainty that cannot be quantified and that can potentially distinguish the disease course of different individuals, even with the same genetic background. In this perspective some stochastic models of RRMS have been presented in the literature. Vélez de Mendizábal et al. [13] presented a Stochastic Differential Equation (SDE) model to reproduce the typical oscillating behavior of RRMS. In [14] the authors presented a (stochastic) agent based model with a more complete description of the entities involved into the disease, including some thoughts about the role of vitamin D [15], and the BBB [16]. However, these models can not be easily re-used because their analysis workflows are often so specific that they can not be directly applied to analyze other contexts different from those for which they were originally developed. To deal with this aspect, in this work we developed a new RRMS model and its associated analysis workflow using *Epimod* [17], a general modeling framework that we recently developed to provide a friendly environment for the modeling and the analysis of complex biological networks. Briefly, the *Epimod* utilization allows us: (1) to use a graphical formalism for the model creation; (2) to implement the analysis workflow exploiting a user-friendly interface based on R language; (3) to achieve the computational reproducibility of the analysis results; (4) to have the possibility to easily create new user-defined analysis workflows.

In detail the new RRMS model extends those proposed in our previous works [18, 19], including new biological components of the immune system relevant to MS disease: (1) the BBB, that mainly has the function of protecting brain tissue from harmful elements present in the blood and that in MS is damaged and crossed by T cells [20]; (2) pro-inflammatory (e.g. IL-17, IFN$\gamma$) and anti-inflammatory (e.g. Interleukin-10 (IL-10)) cytokines, cell signaling molecules that modulate the immune response through the activation of several pathways [20]. Moreover, the model is calibrated by exploring experimental data on 16 subjects, eight MS patients and eight healthy donors, in which the individual variability in terms of number of cells and cytokines production, in the blood and in the cerebrospinal fluid, had been quantified [5]. Then, the model behaviour without and with DAC administration was studied using Stochastic Simulation Algorithm (SSA) [21] method implemented in the R package *Epimod* [17] in order to take into account the stochasticity and the variability of the MS desease.

## Methods

In this section we introduce the high-level Petri Net (PN) extension, called Extended Stochastic Symmetric Net (ESSN) [18], which is used to graphically model our case study. Then, we report how the behaviour of such a system can be efficiently derived by exploiting SSA algorithm. Finally, we briefly introduced the *Epimod* framework exploited in this work to achieve an easy reusable model by other researchers and a reproducible analysis.

### Extended stochastic symmetric net

PNs [22] and their extensions are widely recognized to be a powerful tool for modeling and studying biological systems thanks to their ability of representing systems in a graphical manner and allowing the computation of qualitative and quantitative information about the behavior of these systems. In details PNs are bipartite directed graphs with two types of nodes, the former is called *place* and it is graphically represented as circle, the latter is called *transition* and it is graphically represented as box. Usually, places correspond to the state variables of the system and they can contain tokens encoding the corresponding variable state. Otherwise, transitions represent events that might occur in the system. These two different nodes are connected by *arcs*, which express the relation between states and event occurrences. A specific cardinality (multiplicity) is associated with each arc describing the number of tokens removed from (or added to) the corresponding place upon the firing of the transition the arc is connected to. Graphically it is written beside the arc, but the default value of one is omitted. Then, the number of tokens in each place defines the state of a PN, called *marking*.

We define a transition enabled, if and only if each input place contains a number of colored tokens greater or equal than a given threshold defined by the cardinality of the corresponding input arcs. Thus, the firing of an enabled transition removes a fixed number of tokens from its input places and adds a fixed number of tokens into its output places, according to the cardinality of its input/output arcs.

Among the PNs generalisations proposed in literature, ESSNs [18, 23] extend the PN formalism providing a more compact, parametric, and readable representation of the system. ESSN allow also an easy definition of complex rate functions splitting the set of transitions *T* in two sub-sets $T_{ma}$ and $T_g$. The subset $T_{ma}$ represents the *standard* transitions which fire with a rate following a Mass Action (MA) law. While $T_g$ is represented by the *general* transitions, whose random firing delays have rates that are defined as general real functions that might depend only from the time and the corresponding input places. The *standard* transitions are graphically represented by white bars, while the *general* ones by black bars.

**Fig. 1:**
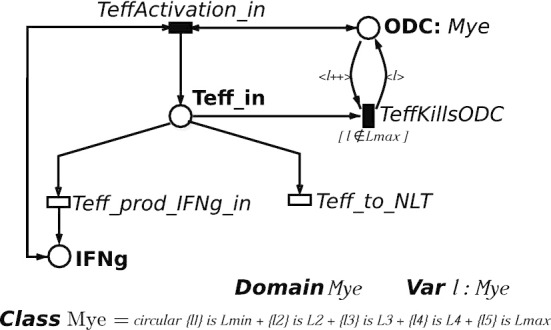
Subnet of ESSN model presented in Fig. 3, representing the Teff activation in CNS, the production of the INFg and the damage the ODCs. *Mye* is circolar color class, representing the myelination levels of ODC cells. This is divided into 5 static subclasses: $L_{min},\ L1,\ L2,\ L3$ and $L_{max}$. *l* is a variable belonging to the color class *Mye*. Moreover the successor operator applied on *l* (i.e. $l++$) is exploited to increase the damage level of an ODC cell. Finally the guard $[ l \notin L_{max}]$ associated with transition *TeffKills ODC* ensures that only ODC cells with damage level different by $L_{max}$ can be attacked by a Teff cell

An example of a simple ESSN is given in Fig. 1 where the activation inside the CNS of the Teff cells due to the myelin sheaths is modeled. Furthermore, the activation velocity depends on the inflammatory cytokines IFN$\gamma$ which are produced by the Teff themselves. This is a portion of the more complex ESSN model represented in Fig. 3, and it is characterized by three places, *Teff_in*, *IFNg_in* and *ODC*, representing the Effector T cells, $\text {IFN}_\gamma$ cells, and the Oligodentrocytes cells, respectively. This model includes: (1) two standard transitions *Teff_to_NLT* and *Teff_prod_IFNg* representing the Teff cells death and the $\text {IFN}_\gamma$ production; and (2) two general transitions *TeffActivation_in*, and *TeffKillsODC* modeling the activation of the Teff cells and the damage of the mylen sheets.

In order to associate colors with the tokens, we have to define a color domain for each place *p*, denoted $cd (p)$. Color domains are defined by the Cartesian product of elementary types called *color classes*, ${\mathcal {C}}=\{ C_1, \dots , C_n \}$, which are finite and disjoint sets, and might be further partitioned into (static) subclasses. They can be ordered (in this case a successor function is defined on the class, denoted by ++, inducing a circular order among the elements in the class), and can be partitioned into (static) subclasses. Similarly, a color domain is associated with transitions and is defined as a set of typed variables where the variables are those appearing in the functions labeling the transition arcs and their types are the color classes. Then, we can define an instance, denoted as $\langle t ,c \rangle$, of a given transition *t* as an assignment (or binding) *c* of the transition variables to a specific color of a proper type. Moreover, we define *guard* a logical expression defined on the color domain of the transition which can be used to define restrictions on the allowed instances of a transition. For instance, in the example in the Fig. 1 the *ODC* color domain is defined by one color class, the myelination levels of ODC cells, named *Mye*. This is divided into 5 static subclasses (i.e. $L_{min},\ L1,\ L2,\ L3$ and $L_{max}$) so that myelination level ranges from an irreversible damage ($L_{min}$, no myelination) to no damages ($L_{max}$, full myelination). So, the color domain of *TeffKillsODC* transition, representing the damage of an ODC cell, is *Mye* and the variable characterizing its input arc is $l \in Mye$.

Each ESSN arc is labeled with an expression defined by the function $I[p,t]$, if the arc connects a place *p* to a transition *t*, or $O[p,t]$ for the opposite direction. The evaluation of $I[p,t]$ (resp. $O[p,t]$), given a legal binding of *t*, provides the multi set of colored tokens that will be withdrawn from (input arc) or will be added to (output arc) the place connected to that arc by the firing of such transition instance. Moreover, we denote with $^{\bullet } {\mathbf {t}}$ and ${\mathbf {t}} ^\bullet$ the set of input and output places, respectively, of the transition *t*. We use the notation *E*(*t*, *m*) to denote the set of all instances of *t* enabled in marking *m*. Where, in the case of ESSN formalism, a transition instance $\langle t ,c \rangle$ is enabled and can fire in an marking *m*, if: (1) its guard evaluated on *c* is true; (2) for each place *p* we have that $I[p,t](c)\le m(p)$, where $\le$ is the comparison operator among multi sets. The firing of the enabled transition instance $\langle t ,c \rangle$ in *m* produces a new marking $m'$ such that, for each place *p*, we have $m'(p)=m(p)+O[p,t](c)-I[p,t](c)$.

In ESSNs each transition is associated with a specific rate, representing the parameter of the exponential distribution that simulates its firing time. So, let define $\hat{m} (\nu )= m(\nu )_{|^{\bullet } {\mathbf {t}} }$ as the subset of the marking $m(\nu )$ concerning only the input places to the transition *t*. Then, the parameter associated with an enabled transition instance $\langle t,c \rangle$ is given by the function

1 $$\begin{aligned} F(\hat{m}(\nu ),t,c,\nu )&:= \left\{ \begin{array}{ll} \varphi (\hat{m}(\nu ),t,c), &{} t\in T_{ma} ,\\ f_{\langle t,c\rangle }( \hat{m}(\nu ),\nu ), &{} t\in T_g, \end{array}\right. \nonumber \\&\qquad f_{\langle t,c\rangle }\in \Omega (t,c) \end{aligned}$$

where $\Omega = \{f_{\langle t,c\rangle }\}_{ t\in T\wedge c \in cd (t)}$ is set grouping all the real functions characterizing the transition speeds $\forall t \in T$, with $f_{\langle t,c\rangle }=\varphi (\cdot ,t,c)$ when $t \in T_{ma}$. Where $\varphi (m(\nu ),t,c)$ is the MA law, i.e.

$$\begin{aligned} \varphi (m(\nu ),t,c)= \omega (t,c) \prod _{\langle p_j,c' \rangle |\ p\in ^{\bullet } {\mathbf {t}} \ \wedge \ c' \in cd (p_j)} m_{p_j,c'}(\nu )^{I[p_j,t](c')[c]} \end{aligned}$$

with $\omega (t,c)$ the rate of the enabled transition instance $\langle t,c\rangle$. Observe that $\varphi (\hat{m}(\nu ),t,c)$ and $f_{\langle t,c\rangle }(\hat{m}(\nu ),\nu )$ can depend only on the time $\nu$ and the marking of the input places of transition *t* at time $\nu$. Stochastic firing delays, sampled from a negative exponential distribution, allow one to automatically derive the underlying Continuous Time Markov Chain (CTMC) that can be studied to quantitatively evaluate the system behaviour [22]. In details, the CTMC state space, $\mathbb {S}$, corresponds to the reachability set of the corresponding ESSN, i.e. all possible markings that can be reached from the initial marking. Thus, the Master equations for the CTMC are defined as follows:

2 $$\begin{aligned} \frac{d\pi (m_i,\nu )}{d\nu } = \sum _{m_k} \pi (m_k,\nu )q_{m_k,m_i} \qquad m_i,m_k \in \mathbb {S} \end{aligned}$$

where $\pi (m_i,\nu )$ represents the probability to be in marking $m_i$ at time $\nu$, and $q_{m_k,m_i}$ the velocity to reach the marking $m_i$ from $m_k$, defined as

$$\begin{aligned} q_{m_k,m_i}= \sum _{ \begin{array}{c} \mathbf{t} \in T \wedge \\ T\langle \mathbf{t} ,\mathbf{c} '\rangle \in E(\mathbf{t} ,m_k)_{|m_i} \end{array}} \,\, F(m_k,\mathbf{t} ,\mathbf{c} ',\nu )(L[p,\mathbf{t} ](\mathbf{c} ')[c]). \end{aligned}$$

where $E(\mathbf{t} ,m_k)_{|m_i}$ is the set of all instances of $\mathbf{t}$ enabled in marking $m_k$ whose firing bring to the marking $m_k$, and $L[p,\mathbf{t} ](\mathbf{c} ')[c] = O[p,\mathbf{t} ](\mathbf{c} ')[c]-I[p,\mathbf{t} ](\mathbf{c} ')[c]$.

In complex systems these Eq. 2 are often computationally intractable, then several techniques, such as the Monte Carlo simulation, can be exploited to study the system taking into account stochasticity.

### Stochastic simulation

Deterministic approximation, in which the system behaviour is approximated by a deterministic model described through an Ordinary Differential Equation (ODE)s system, is one of the most used approach for studying a dynamical system. However, this approach is not able to provide a good approximation of the real system behaviour in those systems in which the randomness plays an important role. To deal with these systems stochastic approaches can be instead exploited.

In 1976 Daniel Gillespie proposed an innovative algorithm, called Stochastic Simulation Algorithm (SSA) [21], to simulate chemical or biochemical systems of reactions. The SSA is an exact stochastic method to simulate chemical systems which provides a set of sample trajectories distributed according to the solution of the Master equations, Eqs. 2. Since this method explicitly simulates all the events that might occur in the system, it becomes often slow when the number of system molecules increased. For this reason several algorithms [24–26] were proposed for obtaining similar approximations as the SSA ones, but with significantly lower computational costs. One of the most common of these approximate simulation algorithms is the $\mathbb {\tau }$**-leaping algorithm** [25]. Indeed by using a Poisson approximation the $\mathbb {\tau }$**-leaping algorithm** can *leap over* many fast reactions and approximate the stochastic behavior of the system very well. Then it provides a natural connection between the SSA in the discrete stochastic regime and the explicit Euler method applied in the continuous deterministic approximation.

### Framework

**Fig. 2:**
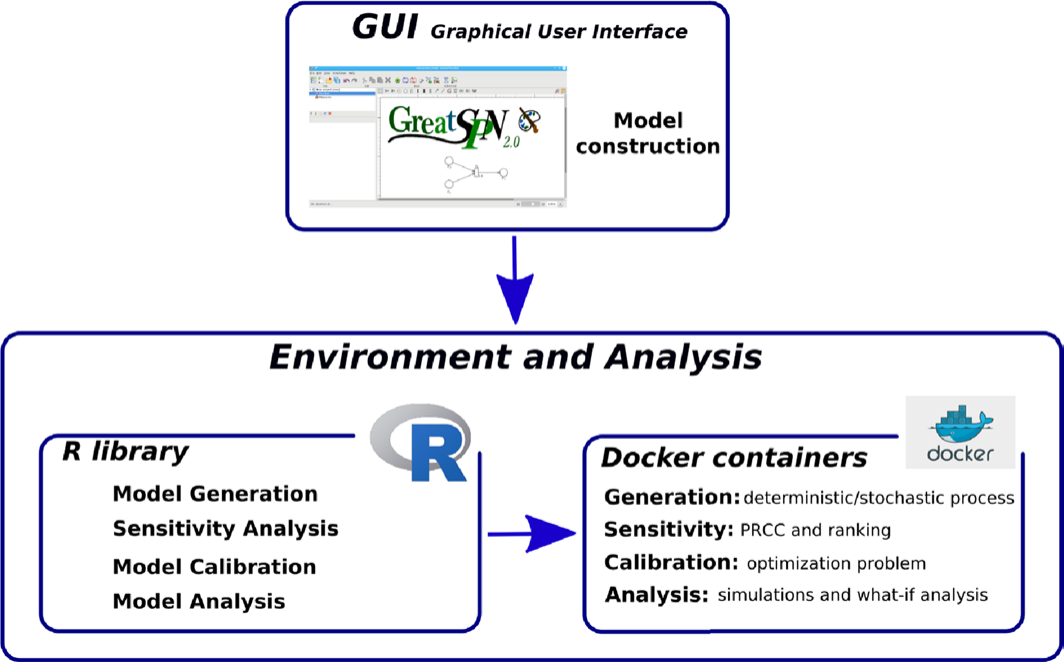
Framework

The creation and analysis of the proposed MS model was carried out using the *Epimod* framework [17]. *Epimod* is an R package providing a modeling framework for the analysis of epidemiological and biological systems, which provides the generation of the deterministic and stochastic process starting from the ESSN graphical formalism, and allows user to carry out the sensitivity, the calibration and the model analysis. In details, by exploiting GreatSPN editor [27], a Java Graphic User Interface (GUI), it is possible to draw and build the ESSN model, and using the generation model function implemented in *Epimod* is possible to easily derive the underlying stochastic and deterministic processes. Successively, the sensitivity analysis reduces the search space associated with each unknown parameter, the calibration analysis adjusts the parameters to obtain the expected behaviour with respect to the available data, and finally the model analysis solves the calibrated model to answer specific questions and to derive new insights. Finally all the implemented analysis techniques were containerized into Docker image to improve the framework portability and to ensure the reproducibility of the derived results. The architecture of this framework is depicted in Fig. 2.

Finally, let us underline that the GreatSPN GUI can be downloaded at https://github.com/greatspn, the R package *Epimod* at https://github.com/qBioTurin/epimod, and all the files exploited through the analysis are freely available at https://github.com/qBioTurin/Multiple-Sclerosis.

## Results

In this section we first describe the computational model developed to study RRMS and then the data exploited to calibrate the model parameters; in particular two parameter configurations are identified for healthy and MS subjects. Finally, we discuss two therapy scenarios in which we investigated the effect of the DAC administration on MS patients. All the analyses are performed on a server with 6 Intel Xeon E5-2650 processors (2.00Ghz, 20MB Cache, 8 Cores) by exploiting *Epimod*.

### Model description

**Fig. 3:**
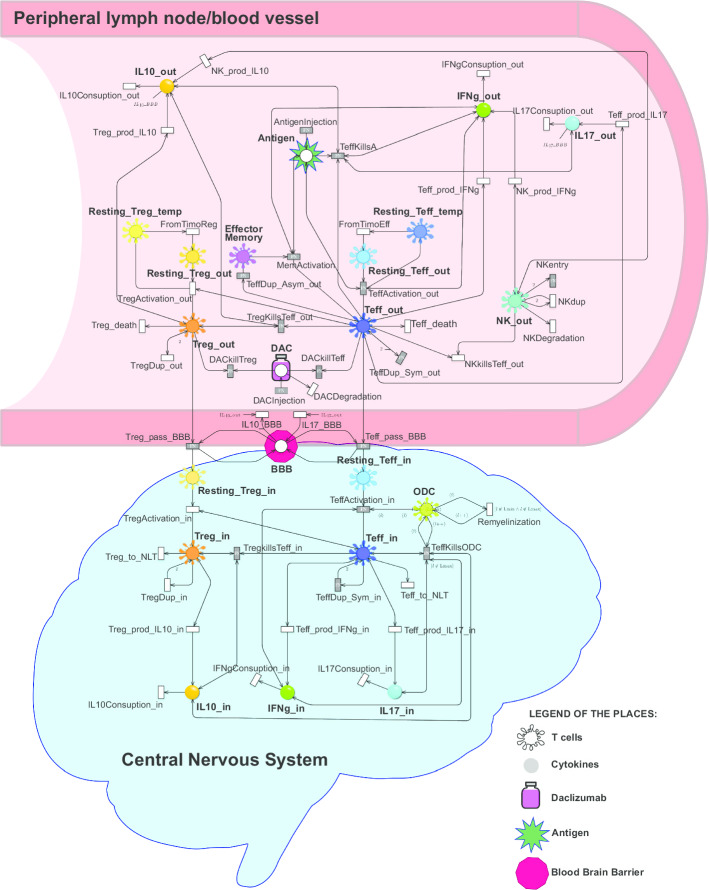
RRMS Model represented by exploiting the ESSN graphical formalism. Each places has on top a sketch of its biological role as described by the legend

The cellular interactions characterizing the immunopathology of RRMS are described by the model showed in Fig. 3. The model consists of 26 places and 55 transitions, of these 40 are standard and 15 general. Observe that to make immediately clear the biological role of each place in the net, we decided to represent them using four different icons whose meaning is reported in the legend of Fig. 3. In details, the model is divided in two compartments: the peripheral lymph node/blood vessel and the CNS. The two compartments interact with each other through the place BBB, as illustrated in Fig. 3. All transitions describing interactions occurring in peripheral lymph node/blood vessel have the suffix “_out” while all transitions taking place in CNS have the suffix “_in”. The Antigen place, simulates the first pathogen infection and then the infection reactivation in the system through the *AntigenInjection* transition.

In the peripheral lymph node compartment of the net the places regarding the Teff cells, including Effector Memory T cells, are represented. After pathogen infection Teff cells can give rise to Teff or Effector memory cell through *TeffDup_Sym_out* and *TeffDup_Asym_out* transitions. Effector memory cells remain in this compartment and are able to respond faster to the infection reactivation. The annihilation of the pathogen by the Teff action is modeled by the transition *TeffKillsA*, while symmetrical and asymmetrical duplication of Teff cells is encoded by transitions *TeffDup_Sym_out* and *TeffDup_Asym_out*. Teff production of the inflammatory cytokines IL-17 and IFN$\gamma$ is represented by the transitions *Teff_prod_IL*$_{17}$ and *Teff_prod_IFNg*, respectively. Inflammatory cytokines are degradated by *IFNgConsumption_out* and *IL17Consuption_out* transitions. Afterwards, the arrival of new resting Treg cells from thymus, the control mechanism of the Treg over the Teff and their activation, proliferation and death are encoded by the transitions *FromTimoReg*, *TregKillsTeff_out*, *TregActivation_out*, *TregDup_out* and *TregDeath*, respectively. Treg production and degradation of the anti-inflammatory cytokines IL-10 are represented by the transitions *Treg_prod_IL*$_{10}$_*out* and *IL10Consuption_out*.

Through the transition *NKarrive* the arrival of a NK cells able to kill self-reactive Teff is simulated. The killing is simulated by the transition *NKKillsTeff_out* and the NK production of IFN$\gamma$ and IL-10 is represented by the transitions *NK_prod_IFNg* and *NK_prod_IL10*, respectively. The death and proliferation of the NK cells are modeled by transition *NKDegradation* and *NKdup*.

The drug administration of daclizumab (DAC) is modeled by transition *DACinjection*, and its degradation by *DACDegradation*. DAC is able to inhibit the expansion of Treg and Teff through the transitions *DACkillTeff* and *DACkillTreg*.

During the relapsing phases of the disease the BBB increases its permeability leading to the passage of Teff and Treg from peripheral blood to CNS . This biological process is encoded by the place *BBB* and transitions *Teff_pass_BBB*, *Treg_pass_BBB*, *IL10_BBB* and *IL17_BBB*.

In the CNS the Resting_Teff_in are activated through the transition *TeffActivation_in*. The ODC damage due to Activated Teff cells is modeled by *TeffKillsODC* whose its fire decreases the myelination levels of ODC s by acting on the colors of the tokens in place ODC^1^. When the myelin level reaches the lowest value, an irreversible damage occurs since the remyelinization of the neurons modeled by the transition *Remyelinization* is no longer possible.

Finally, transition *TeffDup_Sym_in* simulates the Teff cells proliferation. Teff production of the inflammatory cytokines IL-17 and IFN$\gamma$ is represented by the transitions *Teff_prod_IL*$_{17}$_*in* and *Teff_prod_IFNg_in*, respectively. The inflammatory cytokines are degraded by *IFNgConsumption_out* and *IL17Consuption_in* transitions.

Tregs in CNS (*Resting_Treg_in* and *Treg_in*) product and degrade IL-10 thought *Tref_prod_IL10_in* and *IL10Consuption_in*. Their control mechanism on Teff their activation, proliferation and death are encoded by following transitions: *TregkillsTeff_in*, *TregDup_in* and *Treg_to_NLT*.

Parameters, constants and functions are reported in Additional file 1, S1.1.

### Data

For the calibration of the model we used biological data from eight relapsing MS patients and eight healthy donors (Healthy Donors (HD)s). A blood sample and a cerebrospinal fluid (CSF) sample of MS subjects were collected at the onset of the disease. Since CSF withdrawal is an invasive practice, for HDs only a blood sample was obtained. The flow cytometer cell counts have been used to set the initial marking of the net. In details, the cells were isolated from blood and *in-vitro* activated as specified in the methods section of [5]. After 18 hours cells were “tagged” with a mixture of fluorescent antibodies directed towards proteins that identify the culture of interest. Regarding T cells, the combinations of multiple antibodies can be used to identify a specific cellular subtype of interest. In our case, the total of CD4$^+$ T cells / ml of blood or CD4$^+$ T cells / ml of CSF was counted, and, among them, the percentage of cells producing IL-17, IFN$\gamma$ or IL-10 in blood and CSF was identified, see Table 1.

The individual cell counts of MS patients and healthy subjects were used to fit the model parameters.

**Table 1:** Biological data from eight relapsing MS patients and eight HD subjects. Values are expressed as number of cells/mm^3^ 3

Patient ID	IFN$\gamma$-producing cells (blood)	IL17-producing cells (blood)	IL10-producing cells (blood)	IFNg-producing cells (CSF)	IL17-producing cells (CSF)	IL10-producing cells (CSF)
MS1	169.22	30.83	3.52	13.24	3.31	0.06
MS2	21.56	5.33	1.02	5.59	1.71	0.06
MS3	71.10	15.45	2.46	16.10	0.60	0.10
MS4	15.44	21.34	1.54	3.36	0.88	0.04
MS5	148.65	28.66	2.27	17.80	2.87	0.10
MS6	60.31	15.80	4.38	12.27	1.18	0.14
MS7	377.13	53.86	5.13	29.64	2.29	0.11
MS8	156.74	21.87	8.26	10.15	1.64	0.13
mean MS	117	25	3	12.77	1.85	0.09
HD1	42.37	21.19	26.98	0.30	0.30	0.40
HD2	2.40	13.20	16.80	0.80	0.20	1.00
HD3	8.70	7.83	8.18	0.20	0.80	1.00
HD4	69.30	3.15	11.76	1.00	1.00	1.00
HD5	16.15	2.38	8.27	0.10	0.80	0.10
HD6	18.45	1.64	6.48	2.00	1.00	2.00
HD7	63.00	7.88	10.94	0.10	0.10	0.80
HD8	97.27	13.70	12.06	0.20	1.90	1.90
mean HD	42	8	13	0.59	0.76	1.03

**Table 2:** Initial marking of the model

Place	Number of cells/mm^3^	Reference
*Resting_Teff_out*	1689	[18]
*Resting_Treg_out*	63	[18]
*NK_out*	30	[28, 29]
*IL-17_out*	8	[5]
*IL-10_out*	13	[5]
*IFNg_out*	42	[5]
*IL-17_in*	1	[5]
*IL-10_in*	1	[5]
*IFNg_in*	1	[5]
*ODC_Lmax*	500	[18]

### Model calibration

The model calibration was performed on our model to make its behaviours in agreement with the experimental values described above. In details, during the calibration analysis the same initial marking was assumed for both HDs and MS patients, as showed in Table 2, where only the places with initial marking different from zero were reported. Observe that these values correspond to the average values computed considering only the HDs reported in Table 1. Moreover, we considered all the 500 ODC with level *Lmax* of neuronal myelinization at the initial time.

Then, from the model shown in Fig. 3 a system of 26 ODEs composed by 20 unknown parameters was derived. These parameters (reported in Table S1 in the Additional file 1) were characterized by a high uncertainty due to their difficulty of being empirically measured. To identify the parameter values, the ODE system was simulated over 30 days interval assuming a injection of 100 antigen copies at the second day. This analysis lead to identify the best fit characterized by the values described in Sec. Data. In particular, the parameter values for HD and MS patient were identified by minimizing the difference between the numbers of IFN$\gamma$-producing, IL-17-producing IL-10-producing cells in blood and in CSF obtained from the solution of the ODE system and those experimentally measured in average for HDs and MS patients (reported in Table 1) after 18 hours from the antigen injection.

It is worth noting that the two sets of parameter values, respectively for HD and MS patients, obtained by the calibration step, differ only in the values of the parameters $p_{Treg\_Activation}$ and $p_{Teff\_Activation}$ associated with the transitions $TregActivation\_in (\_out)$ and $TeffActivation\_in (\_out)$.

The model behaviours derived by these two parameter configurations are reported in Figure S2 of the Additional file 1. From these plots it is immediately clear that the model outcomes are in agreement with real data and the current knowledge of MS disease, for example the MS patients are characterized by a higher number of irreversibly damaged ODC cells and a more impermeable BBB than HDs.

**Fig. 4:**
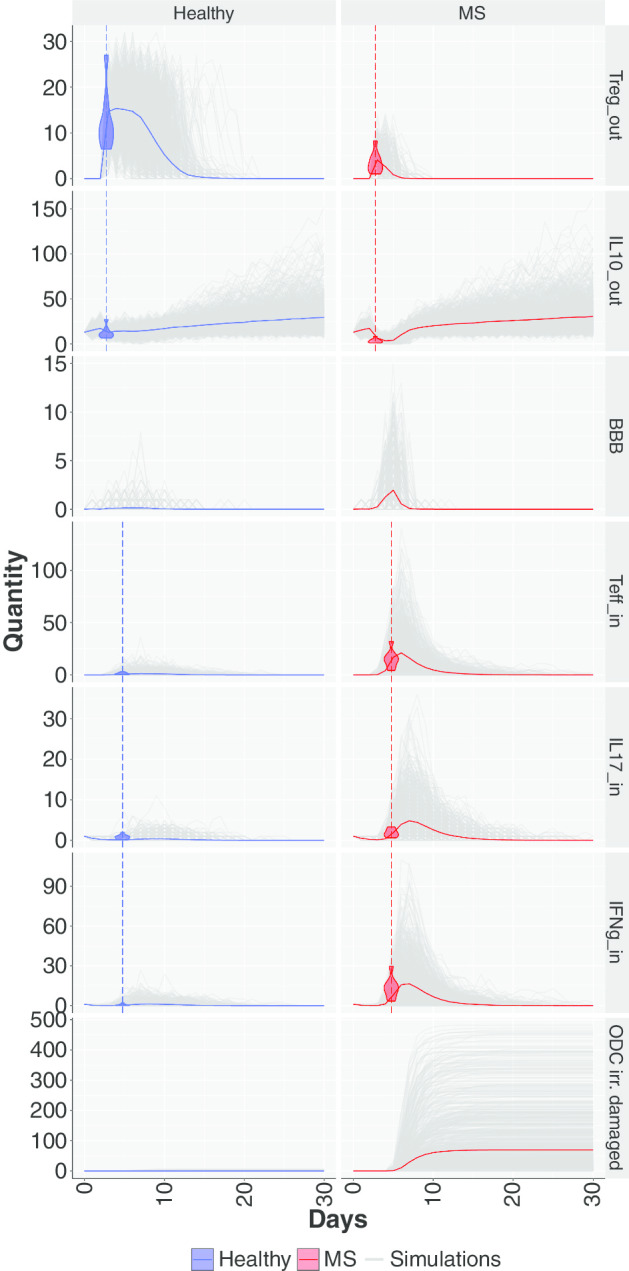
1000 stochastic simulations considering the healthy (first column) and the MS (second column) parameters configuration. The colored bold lines represent the mean traces of the simulations, blue for the healthy and red for the MS scenario. The violin plots are the representation of the real data

*Stochastic simulations* After the model calibration, the two parameter configurations were exploited to investigate the stochastic behaviour of the system using the SSA algorithm. In Fig. 4, 1000 trajectories for each scenario are plotted in grey, while the colored bold line represents the mean trajectory. The distribution of experimental data (Table 1) is represented through violin plots. It is possible to observe that the mean trajectory and the set of 1000 stochastic trajectories are consistent with the experimental measures in both HDs and MS patients. In particular, observing the $1^{st}$ row in Fig. 4, it is possible to appreciate the differences in the number of circulating Treg cells between HD and MS patient. Accordingly, while the antigen is counteracted by the Teff cells, Treg cells try to balance the aggressiveness of the immune system and to maintain the cellular homeostasis by acting as a brake on the inflammatory response producing IL-10and killing Teff cells. In the $2{\rm nd}$ row in Fig.4 the IL-10 dynamics are reported: the MS condition produces less IL-10 with respect to the healthy counterpart. IL-10 cytokines imbalance towards an inflammatory state is reflected in an increased permeability of the BBB. IL-10 contributes to promote BBB integrity, while IL-17 contributes to the damage of BBB, making it permeable to the passage of cells of the immune system and other molecules. Indeed, after less than one week we can observe that in the MS patient BBB has the highest permeability ($3^{rd}$ row in Fig.4) leading to a T cell trafficking in the CNS. This effect is observed in the form of an increased reactivation of Teff cells in the CNS of the MS patient compared to the healthy subject ($4{\rm th}$ row in Fig.4), as well as in the increase in the circulation of pro-inflammatory cytokines produced by the Teff cells circulating in the CNS: IL-17 ($5{\rm th}$ row Fig.4) and IFN$\gamma$ ($6{\rm th}$ row Fig.4). The final result of this pro-inflammatory environment in the CNS is an increased damage to the ODCs, which simulates a neuronal damage to the myelin in the CNS actually observed during the clinical relapse ($7{\rm th}$ row and second column Fig.4). Conversely, ODC damage is not observable in HDs ($7{\rm th}$ row and first column Fig.4). The dynamics of the other cells are reported in Figure S3 of the Additional file 1.

### Daclizumab therapy

To investigate the effect of the DAC therapy in our model calibrated for RRMS patients, we simulated a real scenario with multiple antigen occurrences at different time in two-years time interval. In details we assumed a total of eight injections introducing into the system 100 antigen copies per injection. Most of the MS-related viruses, infect a particular type of immune cells (i.e. the Epstein-Barr Virus (EBV) infects B cells) and once the initial lytic infection is brought under control, the virus persists in the immune repertoire of a subject in a state called “of latency” for the rest of his/her life and is subjected to periodical reactivation [30]. For this reason, we defined a sequence of injections at varying intervals: the first three injections were set at constant time interval, i.e., the $2^{\text {nd}},\ 67^{\text {th}}\ \text {and}\ 127^{\text {th}}$ day, then four consecutive injections were simulated at $295^{\text {th}},\ 300 ^{\text {th}},\ 303^{\text {th}}\ \text {and}\ 307^{\text {th}}$ days, and finally the last injection at $600^{\text {th}}$ days. Two important aspects of the therapy modulation were considered in our simulations: the drug dose and the drug potency. In details, five scenarios characterized with an increasing drug dose (i.e., 1000, 2000, 5000, 10000 and 15000) were analysed. Then for each scenario two drug potencies (i.e. *weak potency* and *strong potency* characterized by *DACkillTreg* and *DACkillTeff* set to 0.01 and 0.03, respectively) are showed for a total of ten different scenarios. These scenarios are proposed in two course of actions: *early regime therapy* in which the DAC administration starts at the first month, and *late regime therapy* in which the DAC administration starts at the sixth month.

**Fig. 5:**
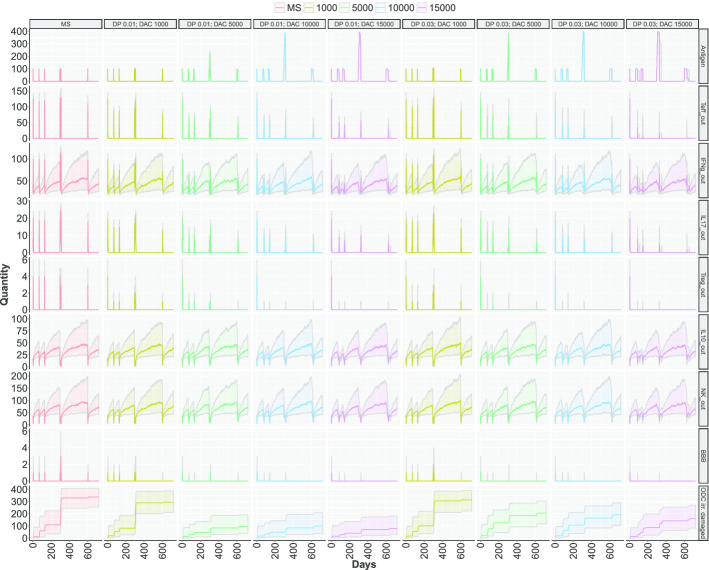
Stochastic simulations considering the **early therapy**. Different colors are associated to quantity of DAC injected for each scenario, from 1000 to 15000 cells. The first two column represent the healthy and MS scenarios. Two drug potencies (called DP) are showed, i.e., 0.01 e 0.03

**Fig. 6:**
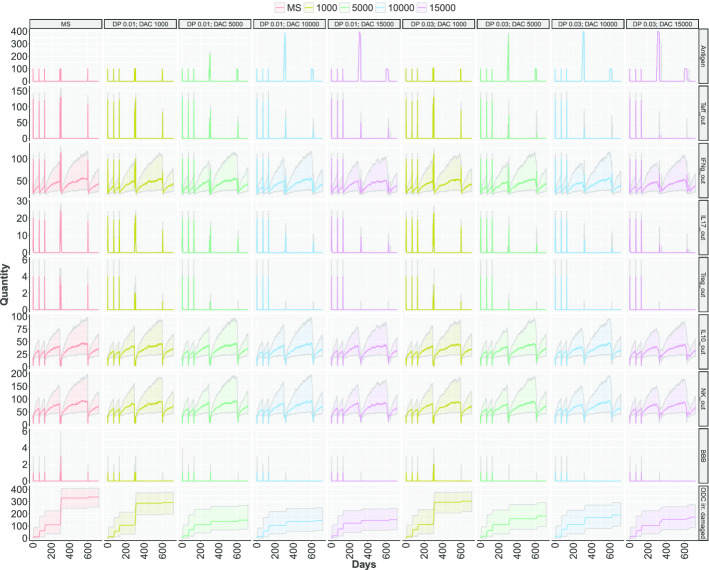
Stochastic simulations considering the **late therapy**. Different colors are associated to quantity of DAC injected for each scenario, from 1000 to 15000 cells. The first two column represent the healthy and MS scenarios. Two drug potencies (called DP) are showed, i.e., 0.01 e 0.03

In Fig. 5 and Fig. 6 the behaviour of the places *Antigen, Teff_out, IFNg_out, IL17_out, Treg_out, IL10_out, NK_out, BBB, and ODC irreversibly damaged* in the early and late regime are reported. In each figure, the columns represent nine scenarios among the twelve described above. Specifically, we decide to omit for clarity the healthy scenario and the ones considering drug dose equals to 2000. The colored bold lines represent the median of 1000 simulations, while the colored areas are the range of the simulations between the first and third quartile. In the Fig.s S4-S5 in the Additional file 1, the complete list of cell types for all the scenarios in both the regime considered are reported.

In both Figs. 5 and 6,the reduction of circulating Teffs and Tregs is visible in all treatment conditions (the $2{\rm nd}$ and $5{\rm th}$ rows), with a remarkable effect at increasing doses. The same effect is visible in the amount of cytokines produced by T cells (the $3^{rd},\ 4{\rm th}$ and $6{\rm th}$ row). Although the DAC is unable to cross the BBB and spread in the CNS, these effects are observable either in the blood and in the CNS (Fig.s S4-S5 in the Additional file 1). This is due to a reduced number of T cells that are not able to effectively reach the CNS and to cause damage. Indeed, the immunosuppressive action of DAC is mediated by the binding to CD25 molecules of IL-2R, present on activated Teff cell and on Treg cells, and results in the inhibition of their proliferation and in the induction of T cell death.

**Fig. 7:**
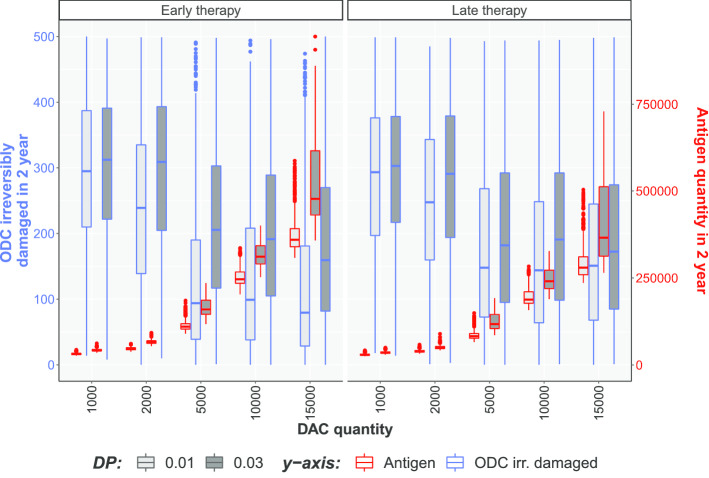
Box-plots representing the 1000 trajectories for each type of (i) therapy (i.e., early and late therapy, and the healthy an MS cases without therapy), (ii) quantity of DAC injected, and (iii) the drug potency. The ODC irreversibly damaged at the end of the two years are plotted with the blue boxplot contour (referring to the left blue y-axis), differently the areas under the antigen curve over the whole two years interval are showed with the red boxplot contour (referring to the right red y-axis)

Figure 7 shows the number of irreversibly damaged ODC (the blue contour boxplot) and the overall antigen concentration (the red contour boxplot) for each scenario. In details, it is showed the difference in the total amount of irreversibly damaged ODCs at the end of the two years, a behavior reflecting the protective effect of DAC therapy on the CNS.

It is observed that for the same dose and potency of DAC, the number of irreversibly damaged ODCs is lower in “early therapy” than in the “late therapy” condition (Fig. 7). Indeed, clinical practice suggests that, in MS, the early intervention reduce neuronal damage and long-term disability [31].

Moreover, an interesting effect of DAC therapy visible by our simulations is that increasing dose and potency of DAC can suppress the immune system by depleting T cells and cytokine diffusion. An overdose of the drug reproduces an immuno-compromised immune system, where T cells are depleted and the antigen persists in the circulation. This is visible in Fig. 7 where the red contours boxplots report the antigen concentration. It is straightforward to see that increasing drug dose is associated with a minor ODC damage at the expense of the antigen annihilation. Moreover, a stronger drug potency is not positive either from the ODC damage and the antigen annihilation point of view. The ideal dose of DAC is a trade-off between antigen annihilation and a reduction in damaged ODC. Drug efficacy must occur with a consideration of the protection from external antigens to minimize the risk of secondary infections - sometimes with serious adverse effects - that often accompany immuno-suppressive therapies in MS [32].

## Discussion

In immunology the use of computational modeling is quite recent, but it is becoming increasingly important. In particular, the computational models can help the researchers to discern between potential right and wrong biological hypotheses, whose confirmation cannot be acquired through *in vivo* or *in vitro* experiments, to find novel treatments, to validate or deduce the mechanisms of actions of existing ones, and to optimize timing and dosage of treatments. The use of such in silico approaches is gaining attention also by regulatory agencies that are starting to foster their application also in the field of personalized-medicine.

In this context, the construction of mathematical models and their solutions remain a challenging tasks mainly due to the lack of general framework easily accessible even by researchers without advanced modelling and mathematical skills. To deal with this aspect, we proposed *Epimod* [17] a general modeling framework specifically developed to provide a friendly environment for the modeling and the analysis of complex biological systems.

In this work, we went a further step forward showing how *Epimod* can be efficiently exploited to study RRMS disease in silico. In details, we extended our previous models [18, 19], including different actors of the pathogenetic mechanisms of MS, as the blood brain barrier and some cytokines produced by self-reactive Teff cells that in MS overcome the BBB and reach the CNS where they damage ODCs [9, 33].

Several evidences suggest the association of viral (and, to a lesser extent, also bacterial) infections with disease onset and relapses leading to hypothesize that RRMS course could be related to a reactivation of a latent infection [33, 34]. EBV, Human Herpes Virus 6 (HHV-6), Varicella Zoster Virus (VZV), and Human Endogenous RetroViruses (HERVs) infections can cause the activation of auto-reactive Teff cells against the CNS [30, 33, 34] through a mechanism called “molecular mimicry”. This is the most frequently discussed mechanism for how viruses or bacteria could induce autoimmunity in MS, that occurs when peptides from pathogens (antigens) share sequence or structural homology with host peptides (self-antigens), in the case of MS with CNS antigens (e.g. Myelin Basic Protein, Myelin oligodendrocyte glycoprotein) [35]. When Teff cells encounter such a foreign peptide, they produce IFN$\gamma$ and IL-17, potent inflammatory mediators able to increase the inflammatory micro-environment. In a healthy host Teff response is tightly regulated by Treg cells to mediate effective host defense against pathogens without causing excessive tissue damage. Furthermore, Treg cells play an important role in maintaining peripheral tolerance to self-antigens. In MS patients, low number and impaired function of Treg cells [7] could explain the massive production of IFN$\gamma$ and IL-17, during the exacerbation phases of MS. In this context, NK cells also give a contribution acting as first-line defense against viruses and bacteria and regulating the auto-reactive Teff cells activity, producing the pro-inflammatory cytokine IFN$\gamma$ and the anti-inflammatory cytokine IL-10 [28, 29].

Interestingly, our healthy and MS model differ just in the two parameters associated to the activation of Teff and and Treg cells. By increasing the Teff and reducing the Treg ones, we were able 1) to fit the real data and 2) to represent the Teff-Treg cell balance characterizing the healthy subject and its unbalance in the MS patient.

Current therapeutic strategies for MS are now focusing on the (i) reduction of the risk of relapses avoiding accumulation of disability and (ii) identification a trade-off between drug efficacy and side effects [31]. DAC was selected for its effects on the depletion of Teff cells, concomitant with a reduction in the number of Treg cells and the expansion of a particular NK cell subset called CD56bright NK cells [12]. In our model, following the administration of DAC, a reduction of Teff cells activation was observed. Furthermore, this reduction was visible for Treg cells too, which normally expose the CD25 on their surface and use it to sequester IL-2 to Teff cells which result in an indirect inhibition mechanism of Teff cells by IL-2 competition [36]. On the other hand, NK cells, trying to make up for the lack of T cells, undergo a selective expansion, as expected [12].

We observed that a dose below 1,000 DAC molecules leads to an insufficient protection from relapses (i.e. ODC damage), while a dose above 10,000 DAC molecules totally depletes the pool of T cells, impairing the mechanisms of protection from external antigens, as shown by the increase of antigen in the system. The right dosage in our model should be set to a value between 1,000 and 10,000 DAC molecules, corresponding to the actual therapeutic range of 150-300 mg per four weeks consistently with the idea that immunosuppressive therapies cannot totally imbalance immune cell homeostasis, as a minimal level of immunesurveillance should be always mantained in order to clear phatogens. Moreover, simulation results about to the comparison between early and late interventions support the current guidelines of therapy for MS, in which is suggested that early intervention is crucial for minimize the accumulation of disability [31].

However, some aspects of our modeling framework can be further improved and others have to be implemented in order to assert its reliability in representing the disease course even in presence of other disease modifying therapies. More specifically, the success of therapies tailored against B cells in MS (e.g. ocrelizumab) has shown how B cells can contribute to the pathogenesis of MS [37], that for a long time was erroneously attributed to T cells only. Also DAC itself has a reduction effect on the B cell population in MS [37]. It is worth mentioning that the role of B cells in MS seems to relate on both antibody-dependent and antibody-independent functions of these cells [30]. Antibody-independent functions are represented by the presentation of the antigen to T cells and modulation of T cell function by secreting pathogens and / or protective cytokines in the CNS [37]. Indeed, the secondary reactivation in the CNS is known to be mediated by contact between antigen-presenting cells (e.g. B cells) and self-reactive T cells. These important aspects of the relationship between B and T cells in MS pathogenesis are worth to be investigated. Moreover, the inclusion of different components and cell types in the model will give us the opportunity to model the action of other therapies besides DAC.

## Conclusions

In this paper we proposed an in silico model based on high level Petri Net formalism exploiting *Epimod* framework. The model has proved to be able to reproduce in silico (i) the fine balance between immune activation and regulation during the RRMS disease course and (ii) the effects of DAC which is a specific therapy tailored against the CD25, a molecule expressed on T cells in order to reduce their activation. Even if the model is not complete, it takes into account the main actors at the basis of MS immune pathogenic process starting from the patients quantification of self-reactive Teff cells and Treg cells. Self-reactive Teff cells produce pro-inflammatory cytokines IL-17 and IFN$\gamma$, which increase during the relapses and are able to damage, first the BBB, and then ODCs whereas Treg cells display an impaired function. Of note, our model confirmed the importance of a timely intervention on the disease course.

## Supplementary information

**Additional file 1.** **S1)** Mathematical details, **S2)** Additional figures.

## Data Availability

All data generated and analyzed during this study are included in this article and its Additional file 1. Moreover, all the R files and the GreatSPN file of the net are freely available at https://github.com/qBioTurin/Multiple-Sclerosis.

## Footnotes

1 Observe that the tokens in ODC are colored according to color class *Mye * that is divided into five static subclasses ranging from *Lmin* (no myelination) to *Lmax* (full myelination) of the color class

## References

[1] Dutta R, Trapp BD (2011). Mechanisms of neuronal dysfunction and degeneration in multiple sclerosis. Prog Neurobiol.

[2] Langrish CL, Chen Y, Blumenschein WM, Mattson J, Basham B, Sedgwick JD, McClanahan T, Kastelein RA, Cua DJ (2005). Il-23 drives a pathogenic t cell population that induces autoimmune inflammation. J Exp Med.

[3] Kebir H, Kreymborg K, Ifergan I, Dodelet-Devillers A, Cayrol R, Bernard M, Giuliani F, Arbour N, Becher B, Prat A (2007). Human th 17 lymphocytes promote blood-brain barrier disruption and central nervous system inflammation. Nat Med.

[4] Durelli L, Conti L, Clerico M, Boselli D, Contessa G, Ripellino P, Ferrero B, Eid P, Novelli F (2009). T-helper 17 cells expand in multiple sclerosis and are inhibited by interferon-β. Ann Neurol.

[5] Rolla S, Bardina V, De Mercanti S, Quaglino P, De Palma R, Gned D, Brusa D, Durelli L, Novelli F, Clerico M (2014). Th22 cells are expanded in multiple sclerosis and are resistant to ifn-β. J Leukocyte Biol.

[6] Tzartos JS, Friese MA, Craner MJ, Palace J, Newcombe J, Esiri MM, Fugger L (2008). Interleukin-17 production in central nervous system-infiltrating t cells and glial cells is associated with active disease in multiple sclerosis. Am J Pathol.

[7] Zozulya AL, Wiendl H (2008). The role of regulatory t cells in multiple sclerosis. Nat Clin Pract Neurol.

[8] Compston A, Coles A (2008). Multiple sclerosis. Lancet (Lond, Engl).

[9] Dendrou CA, Fugger L, Friese MA (2015). Immunopathology of multiple sclerosis. Nat Rev Immunol.

[10] Ahmed SI, Aziz K, Gul A, Samar SS, Bareeqa SB (2019). Risk of multiple sclerosis in epstein-barr virus infection. Cureus.

[11] Shirley M (2017). Daclizumab: a review in relapsing multiple sclerosis. Drugs.

[12] Gold R, Radue E-W, Giovannoni G, Selmaj K, Havrdova EK, Montalban X, Stefoski D, Sprenger T, Robinson RR, Fam10 S. et al. Long-term safety and efficacy of daclizumab beta in relapsing–remitting multiple sclerosis: 6-year results from the selected open-label extension study. J Neurol. 2020. 10.1007/s00415-020-09835-y PMC7501126 32451615

[13] Vélez de Mendizábal N, Carneiro J, Solé RV, Goñi J, Bragard J, Martinez-Forero I, Martinez-Pasamar S, Sepulcre J, Torrealdea J, Bagnato F, Garcia-Ojalvo J, Villoslada P (2011). Modeling the effector-Regulatory T cell cross-regulation reveals the intrinsic character of relapses in Multiple Sclerosis. BMC Syst Biol.

[14] Pennisi M, Rajput AM, Toldo L, Pappalardo F (2013). Agent based modeling of treg-teff cross regulation in relapsing-remitting multiple sclerosis. BMC Bioinf.

[15] Pappalardo F, Pennisi M, Rajput A-M, Chiacchio F, Motta S. Relapsing-remitting multiple scleroris and the role of vitamin D: an agent based model. In: ACM-BCB, 2014; pp. 744–748

[16] Pennisi M, Russo G, Motta S, Pappalardo F (2015). Agent based modeling of the effects of potential treatments over the blood-brain barrier in multiple sclerosis. J Immunol Methods.

[17] Castagno P, Pernice S, Ghetti G, Povero M, Pradelli L, Paolotti D, Balbo G, Sereno M, Beccuti M (2020). A computational framework for modeling and studying pertussis epidemiology and vaccination. BMC Bioinf.

[18] Pernice S, Pennisi M, Romano G, Maglione A, Cutrupi S, Pappalardo F, Balbo G, Beccuti M, Cordero F, Calogero RA (2019). A computational approach based on the colored petri net formalism for studying multiple sclerosis. BMC Bioinf.

[19] Pernice S, Beccuti M, Do’ P, Pennisi M, Pappalardo F. Estimating daclizumab effects in multiple sclerosis using stochastic symmetric nets. In: IEEE International Conference on Bioinformatics and Biomedicine, BIBM 2018, Madrid, Spain, December 3–6, 2018, 2018; pp. 1393–1400.

[20] Passos GRD, Sato DK, Becker J, Fujihara K. Th17 cells pathways in multiple sclerosis and neuromyelitis optica spectrum disorders: pathophysiological and therapeutic implications. Med Inflam. 2016;2016:10.1155/2016/5314541 PMC4749822 26941483

[21] Gillespie DT (1977). Exact stochastic simulation of coupled chemical reactions. J Phys Chem.

[22] Marsan MA, Balbo G, Conte G, Donatelli S, Franceschinis G (1995). Modelling with generalized stochastic petri nets.

[23] Pernice S, Follia L, Balbo G, Sartini G, Totis N, Lió P, Merelli I, Cordero F, Beccuti M. Integrating petri nets and flux balance methods in computational biology models: a methodological and computational practice. Fund Inf, To be published; 2019.

[24] Gibson MA, Bruck J (2000). Efficient exact stochastic simulation of chemical systems with many species and many channels. J Phys Chem A.

[25] Gillespie DT (2001). Approximate accelerated stochastic simulation of chemically reacting systems. J Chem Phys.

[26] Cao Y, Li H, Petzold L (2004). Efficient formulation of the stochastic simulation algorithm for chemically reacting systems. J Chem Phys.

[27] Amparore EG, Balbo G, Beccuti M, Donatelli S, Franceschinis G. 30 years of GreatSPN. In: Principles of performance and reliability modeling and evaluation, pp. 227–254. Springer, Berlin; 2016

[28] Poli A, Michel T, Thérésine M, Andrès E, Hentges F, Zimmer J (2009). Cd56bright natural killer (nk) cells: an important nk cell subset. Immunology.

[29] Laroni A, Uccelli A (2020). Cd56bright natural killer cells: a possible biomarker of different treatments in multiple sclerosis. J Clin Med.

[30] Bar-Or A, Pender MP, Khanna R, Steinman L, Hartung H-P, Maniar T, Croze E, Aftab BT, Giovannoni G, Joshi MA (2020). Epstein-barr virus in multiple sclerosis: theory and emerging immunotherapies. Trends Mol Med.

[31] Montalban X, Gold R, Thompson AJ, Otero-Romero S, Amato MP, Chandraratna D, Clanet M, Comi G, Derfuss T, Fazekas F (2018). Ectrims/ean guideline on the pharmacological treatment of people with multiple sclerosis. Multiple Scler J.

[32] Rolla S, Maglione A, De Mercanti SF, Clerico M (2020). The meaning of immune reconstitution after alemtuzumab therapy in multiple sclerosis. Cells.

[33] Steelman AJ (2015). Infection as an environmental trigger of multiple sclerosis disease exacerbation. Front Immunol.

[34] Oskari Virtanen J, Jacobson S (2012). CNS & neurological disorders-drug targets (formerly current drug targets-CNS & neurological disorders). Viruses Multiple Scler.

[35] Libbey JE, McCoy LL, Fujinami RS (2007). Molecular mimicry in multiple sclerosis. Int Rev Neurobiol.

[36] Sojka DK, Huang Y-H, Fowell DJ (2008). Mechanisms of regulatory t-cell suppression-a diverse arsenal for a moving target. Immunology.

[37] Gharibi T, Babaloo Z, Hosseini A, Marofi F, Ebrahimi-kalan A, Jahandideh S, Baradaran B. The role of b cells in the immunopathogenesis of multiple sclerosis. Immunology. 2020;. 10.1111/imm.13198 PMC7370136 32249925

