# Supplementary material for: Computational modeling of the immune response in multiple sclerosis using epimod framework
Source: BMC Bioinformatics. 2020 Dec 14;21(Suppl 17):550. doi: 10.1186/s12859-020-03823-9 (PMC7734848; doi:10.1186/s12859-020-03823-9)
Supplement: Supplementary file 1 — Additional file 1. S1) Mathematical details, S2) Additional figures. [file 12859_2020_3823_MOESM1_ESM.pdf]

S1 Model

In this section we reported the functions associated with the general transitions of the Extended Stochastic Symmetric Nets (ESSNs) [1, 2] model , and successively the constants values and parameters exploited through the analysis.

S1.1 General transitions

Let us recall that by the definition of the ESSN formalism, transitions which do not follow the Mass Action (MA) law are modelled as general transitions because. In details the speed of general transition $t \in T_g$ is defined by a function $f_{\langle t,c \rangle}(\hat{x}(\nu), \nu)$, where $\hat{x}(\nu)$ represents the vector of the average number of tokens for all the transition input places at time ν and $\langle t, c \rangle$ is the instance transition. Given that the place *ODC* is the only place with an associated color domain, just the functions associated with transitions connected with this place will depend on the color instance c, for the other cases the function will be defined as $f_t(\hat{x}(\nu), \nu)$.

In the ESSN representing the Relapsing-Remitting Multiple Sclerosis (RRMS) introduced in the main paper, see Fig. S1, the general transitions are 15 modeling the i) the killing of a cell, e.g., *TregKillsTeff_in* or *TeffKillsODC*, ii) the entry of Natural Killer (NK) cells into the system, *NKentry*, iii) the activation of T cells, e.g., *TeffActivation_in*, iv) the duplication of a cell, e.g., such as *TeffDup_in*, and v) the injections of the DAC and Antigen into the system, i.e., *DACInjection* and *AntigenInjection*.

Duplication. Considering the Teff duplication event we have to distinguish two possible cases: 1) the Teff symmetric duplication with probability $\rho^{dup} = 2/3$ and a Teff asymmetric duplication, implying the T Memory effector differentiation, with probability $\rho^{mem} = 1 - \rho^{dup}$. This is modeled exploiting three different transitions: *TeffDup_Sym_out(_in)* and *TeffDup_Asym_out*. In the CNS we model only the symmetric differentiation. The asymmetric differentiation give rise to the production of Effector memory cells. These last remain in blood circulation and are able to respond faster to antigen stimulation.The asymmetric differentiation occurs in peripheral blood circulation, then the produced effector memory cells can migrate to all other tissues (e.g. CNS).

So let define

$$r^{in}_{dup} = x_{Teff\_in}(\nu) * p_{Teff\_Dup}$$
$$r^{out}_{dup} = x_{Teff\_out}(\nu) * p_{Teff\_Dup}$$

then these two transitions are defined as:

$$f_{TeffDup\_Sym\_in}(\hat{x}(\nu), \nu) = \rho^{dup} * r^{in}_{dup}$$
$$f_{TeffDup\_Sym\_out}(\hat{x}(\nu), \nu) = \rho^{dup} * r^{out}_{dup}$$

and

$$f_{TeffDup\_Asym\_out}(\hat{x}(\nu), \nu) = \rho^{mem} * r^{out}_{dup},$$

Where p_{Teff_Dup} is the constant Teff duplication rate, see Table S1, and $\hat{x}(\nu) = x_{Teff\_in}$ when the transition considered is the *TeffDup_Sym_in*, otherwise $\hat{x}(\nu) = x_{Teff\_out}$.

Activation. The *TeffActivation_out* and *TeffActivation_in* transitions model the activation of the Teff cells in the peripheral lymphonode/blood vessel (*_out*) and the Central Nervous System (CNS) (*_in*).
The activation of the Teff cells is modeled by the following functions

$$\begin{aligned} f_{TeffActivation\_out}(\hat{x}(\nu), \nu) &= p_{TeffActivation} * x_{RestingTeff\_out}(\nu) * x_{Antigen}(\nu) \\ &\quad * (0.5 + exp(-x_{IFNg\_out}(\nu)/Cifn)); \\ f_{\langle TeffActivation\_in,l \rangle}(\hat{x}(\nu), \nu) &= p_{TeffActivation} * x_{RestingTeff\_in}(\nu) * x_{ODC\_l}(\nu) \\ &\quad * (0.5 + exp(-x_{IFNg\_in}(\nu)/Cifn)), \end{aligned}$$

where $p_{TeffActivation}$ is the constant Teff activation rate, see Table S1, and $\hat{x}(\nu) = \{x_{RestingTeff\_out}, x_{Antigen}, x_{IFNg\_out}\}$ when the transition considered is the *TeffActivation_out*, otherwise $\hat{x}(\nu) = \{x_{RestingTeff\_in}, x_{ODC\_l}, x_{IFNg\_in}\}_{l \in cd(ODC)}$. Let us note that the term $(0.5 + exp(-x_{IFNg\_in(\_out)}(\nu)/Cifn))$ is a coefficient varying in $[0.5, 1.5]$ respectively to the concentration of INF_{γ}, more is present into the system slower is the velocity. In particular when there is no INF_{γ} then to the velocity is associated the highest value, (i.e., 1.5). otherwise it decreases until 0.5 .

Similarly, the transition *MemActivation* modeling the activation of T Memory effectors only in the peripheral lymphonode/blood vessel is defined as

$$f_{MemActivation}(\hat{x}(\nu), \nu) = \begin{cases} 0 & \nu < t_{2inj}, \\ r_{MemA} * x_{EffectorMemory}(\nu) & \nu \geq t_{2inj}, \end{cases}$$

where

$$r_{MemA} = 2 * p_{TeffActivation} * x_{Antigen}(\nu) * (0.5 + exp(-x_{IFNg\_out}(\nu)/Cifn)),$$

and t_{2inj} is the time corresponding to the second antigen injection. We are considering the velocity of this transition as zero $\forall \nu < t_{2inj}$, since we are assuming that the T Memory effectors start to react after the first virus occurrence, with twice the velocity of the Teff cells (for this reason we have $2 * p_{TeffActivation}$).

NK entry. The *NKentry* transition keeps in a constant range around 30 ([3, 4]) the number of NK_out. It is defined by the following function:

$$f_{NKentry}(\hat{x}(\nu), \nu) = \begin{cases} 0 & x_{NK\_out}(\nu) > 30, \\ 0.267 * (30 - x_{NK\_out}(\nu)) & x_{NK\_out}(\nu) \leq 30. \end{cases}$$

Killing. The general transitions modeling the killing of specific cell are the following:

- the *TregKillsTeff_out* and *TregKillsTeff_in* modeling the controlling action of the Treg over the Teff cells. These are defined as follows:

$$\begin{aligned} f_{TregKillsTeff\_out}(\hat{x}(\nu),\nu) &= p_{TregKillsTeff} * x_{Treg\_out}(\nu) * x_{Teff\_out}(\nu) \\ &\quad * (1 - exp(-x_{IL10\_out}(\nu)/cIL10)), \\ f_{TregKillsTeff\_in}(\hat{x}(\nu),\nu) &= p_{TregKillsTeff} * x_{Treg\_in}(\nu) * x_{Teff\_in}(\nu) \\ &\quad * (1 - exp(-x_{IL10\_in}(\nu)/cIL10)), \end{aligned}$$

  where the coefficient $(1-exp(-x_{IL10\_in}(\nu)/cIL10))$ varies in [0,1] representing the Treg cell need fo te IL-10 for suppression of the Teff cells. Indeed, with an increasing number of IL-10 (i.e., the coefficient goes to 1) the transition velocity increases, otherwise it decreases.
- The *TeffkillsA* and *TeffKillsODC* modeling the annihilation of the pathogen by the Teff action and the ODC damage due to Activated Teff cells, respectively, are defined as follows:

$$\begin{aligned} f_{TeffkillsA}(\hat{x}(\nu),\nu) &= p_{TeffkillsA} * \Theta^{out}(\nu) * x_{Antigen}(\nu) * x_{Teff\_out}(\nu), \\ f_{\langle TeffKillsODC,l\rangle}(\hat{x}(\nu),\nu) &= p_{TeffKillsODC} * \Theta^{in}(\nu) * x_{ODC_l}(\nu) * x_{Teff\_in}(\nu), \end{aligned}$$

  with $l \in cd(ODC)$, Let define $\Theta^{in(/out)}$ as a coefficient varying in $[0.5, 1.5]$ which takes in account the pro- (IL-17, IFNg) and anti-inflammatory (IL-10) cytokines in order to increase the velocity of the Teff action when more pro-inflammatory cytokines are present into the system, or decrease it otherwise. These coefficients are defined as follows:

$$\begin{aligned} \Theta^{out} &= 1 + 0.5 * \frac{x_{IL17\_out}(\nu) + x_{IFNg\_out}(\nu) - x_{IL10\_out}(\nu)}{x_{IL17\_out}(\nu) + x_{IFNg\_out}(\nu) - x_{IL10\_out}(\nu)}, \\ \Theta^{in} &= 1 + 0.5 * \frac{x_{IL17\_in}(\nu) + x_{IFNg\_in}(\nu) - x_{IL10\_in}(\nu)}{x_{IL17\_in}(\nu) + x_{IFNg\_in}(\nu) - x_{IL10\_in}(\nu)}. \end{aligned}$$

- Finally the Daclizumab action to control the Treg and Teff cells spreading is modeled by the transitions *DACkillTeff* and *DACkillTreg*, whose functions are defined as follows:

$$\begin{aligned} f_{DACkillTeff}(\hat{x}(\nu),\nu) &= p_{DACkill} * \frac{x_{Treg\_out}(\nu)}{x_{Treg\_out}(\nu) + x_{Teff\_out}(\nu)} * x_{DAC}(\nu) \\ f_{DACkillTreg}(\hat{x}(\nu),\nu) &= p_{DACkill} * \frac{x_{Teff\_out}(\nu)}{x_{Treg\_out}(\nu) + x_{Teff\_out}(\nu)} * x_{DAC}(\nu) \end{aligned}$$

where the coefficient $\dfrac{1}{x_{Treg\_out}(\nu) + x_{Teff\_out}(\nu)}$ scales the velocity with respect to the number of Teff and Treg cells.

The parameters $p_{\text{Transition name}}$ are defined in Table S1.

Injections. The *AntigenInjection* and *DACinjection* transitions inject into the system specific quantities of Antigen and Daclizumab respectively at fixed time points. These transitions are modeled by the occurrence of discrete events during the simulation which modify the model marking in specific time points (feature implemented in the *Epimod* Package [5]).

S1.2 Model parameters

Table S1 List of the calibrated parameters to fit with the real data, in both the healthy and MS patient.

Parameter	Transition	Healthy Value	MS Value
$p_{Teff\_Activation}$	$TeffActivation\_out$ $MemActivation$ $TeffActivation\_in\_l\_le1$ $TeffActivation\_in\_l\_le2$ $TeffActivation\_in\_l\_le3$ $TeffActivation\_in\_l\_le4$ $TeffActivation\_in\_l\_le5$	0.015	0.018
$p_{Treg\_Activation}$	$TregActivation\_in$ $TregActivation\_out$	4e-04	7e-05
p_{Treg_Dup}	$TregDup\_in$ $TregDup\_out$	0.006	–
p_{Teff_Dup}	$TeffDup\_Asym\_out$ $TeffDup\_Sym\_in$ $TeffDup\_Sym\_out$	0.04	–
$p_{TeffKillsODC}$	$TeffKillsODC\_l\_le1$ $TeffKillsODC\_l\_le2$ $TeffKillsODC\_l\_le3$ $TeffKillsODC\_l\_le4$	6e-04	–
p_{TrkTe}	$TregKillsTeff\_in$ $TregKillsTeff\_out$	0.02	–
p_{TekA}	$TeffkillsA$	6e-04	–
$p_{Pass\_BBB\_treg}$	$Treg\_pass\_BBB$	0.45	–
$p_{Pass\_BBB\_teff}$	$Teff\_pass\_BBB$	0.005	–
$p_{NKkillsTeff}$	$NKkillsTeff\_out$	0.01	–
$p_{NK\_prod\_IFNg}$	NK_prod_IFNg	0.03	–
$p_{NK\_prod\_IL10}$	NK_prod_IL10	0.045	–
p_{IL17_BBB}	$IL17\_BBB$	0.0115	–
p_{IL10_BBB}	$IL10\_BBB$	0.0765	–
$p_{Remyelinization}$	$Remyelinization\_l\_le2$ $Remyelinization\_l\_le3$ $Remyelinization\_l\_le4$	0.01	–
$p_{IL10Consuption}$	$IL10Consuption\_out$ $IL10Consuption\_in$	0.09	–
$p_{IL17Consuption}$	$IL17Consuption\_out$ $IL17Consuption\_in$	0.03	–
$p_{IFNgConsuption}$	$IFNgConsuption\_out$ $IFNgConsuption\_in$	0.05	–
$Cifn$	as $p_{Teff\_Activation}$	20	–
$CIL10$	as $p_{TregKillsTeff}$	10	–

Table S2 List of the fixed parameters.

Transition	Rate value
FromTimoREG	0.317
FromTimoEFF	0.296
NKdup	$1/24h^{-1}$
NKDegradation	$1/24h^{-1}$
Teff_death	$1/24h^{-1}$
Teff_to_NLT	$1/24h^{-1}$
Treg_death	$1/24h^{-1}$
Treg_to_NLT	$1/24h^{-1}$
Treg_prod_IL10[1]	0.05556
Teff_prod_IL17	0.00895
Teff_prod_IFNg	0.0466
DACDegradation [2]	0.001444057

S1.3 Data information

Referring to the Table 2 in the main paper, we related those numbers to 1 ul and identified the values to be included in our model. In particular, we infer the numbers of T effector cells from the sum of IFNg values and IL-17 values, and the the number of regulatory T cells from IL-10 values. Differently, to attribute a value for circulating cells in the CNS in healthy subjects we identified a threshold value from the literature and then attributed random values below this threshold [7].

S2 Supplementary figures

Author details

References

1. Pernice, S., Follia, L., Balbo, G., Sartini, G., Totis, N., Lió, P., Merelli, I., Cordero, F., Beccuti, M.: Integrating petri nets and flux balance methods in computational biology models: a methodological and computational practice. Fundamenta Informaticae, To be published (2019)
2. Pernice, S., Pennisi, M., Romano, G., Maglione, A., Cutrupi, S., Pappalardo, F., Balbo, G., Beccuti, M., Cordero, F., Calogero, R.A.: A computational approach based on the colored petri net formalism for studying multiple sclerosis. BMC bioinformatics **20**(6), 1–17 (2019)
3. Poli, A., Michel, T., Thérésine, M., Andrès, E., Hentges, F., Zimmer, J.: Cd56bright natural killer (nk) cells: an important nk cell subset. Immunology **126**(4), 458–465 (2009)
4. Laroni, A., Uccelli, A.: Cd56bright natural killer cells: A possible biomarker of different treatments in multiple sclerosis. Journal of Clinical Medicine **9**(5), 1450 (2020)
5. Castagno, P., Pernice, S., Ghetti, G., Povero, M., Pradelli, L., Paolotti, D., Balbo, G., Sereno, M., Beccuti, M.: A computational framework for modeling and studying pertussis epidemiology and vaccination, (2020)
6. Keizer, R.J., Huitema, A.D., Schellens, J.H., Beijnen, J.H.: Clinical pharmacokinetics of therapeutic monoclonal antibodies. Clinical pharmacokinetics **49**(8), 493–507 (2010)
7. Deisenhammer, F., Zetterberg, H., Fitzner, B., Zettl, U.K.: The cerebrospinal fluid in multiple sclerosis. Frontiers in immunology **10**, 726 (2019)

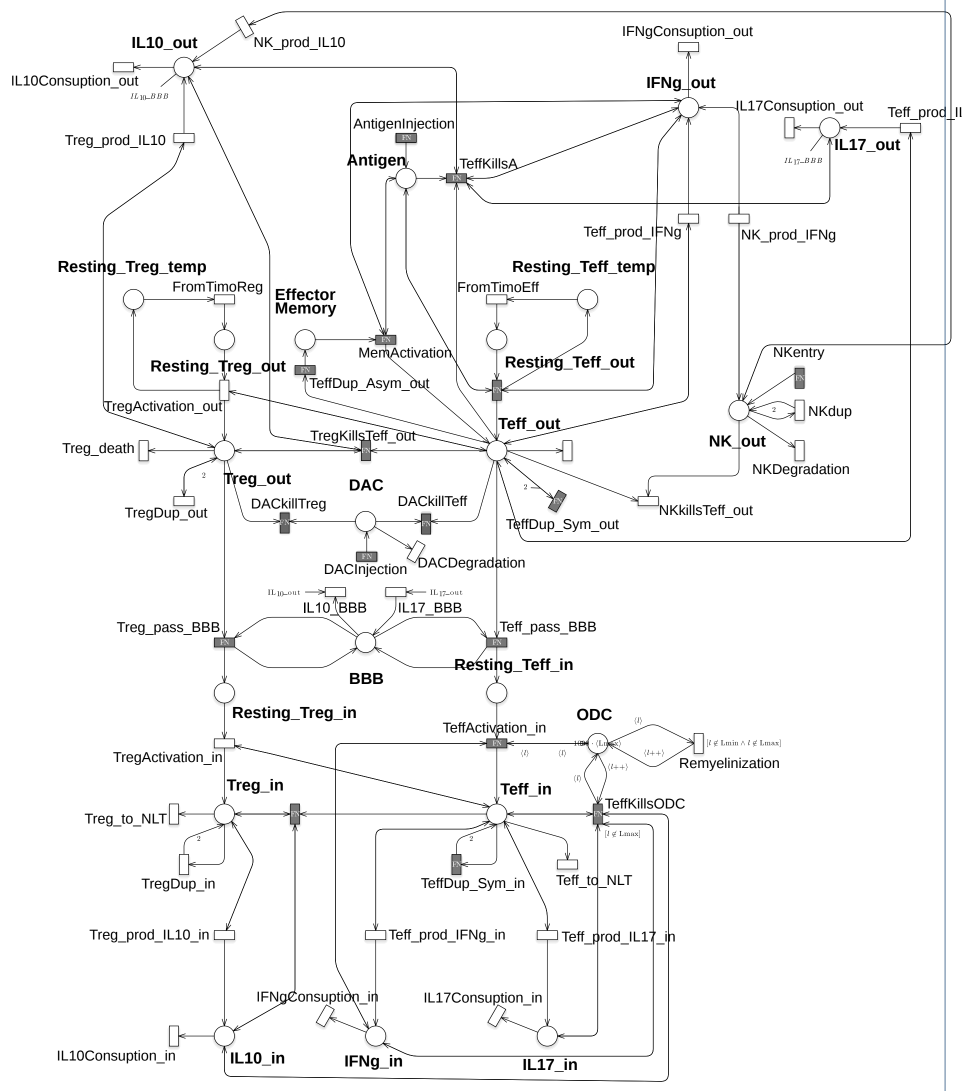

Figure S1 RRMS model represented by exploiting the ESSN graphical formalism. Name of the places are represented in bold text

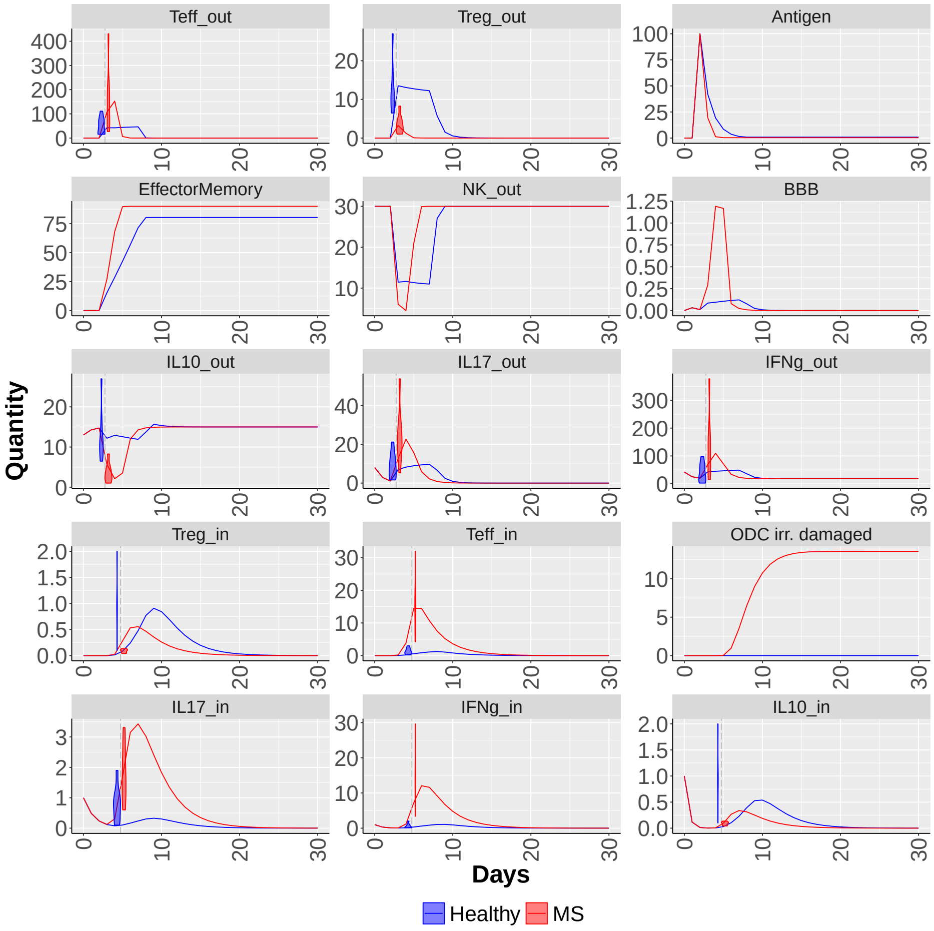

Figure S2 Deterministic solution of the ODEs system considering both the parameters combinations, red the MS patient and blue the healthy individual. The violin plots are the representation of the real data.

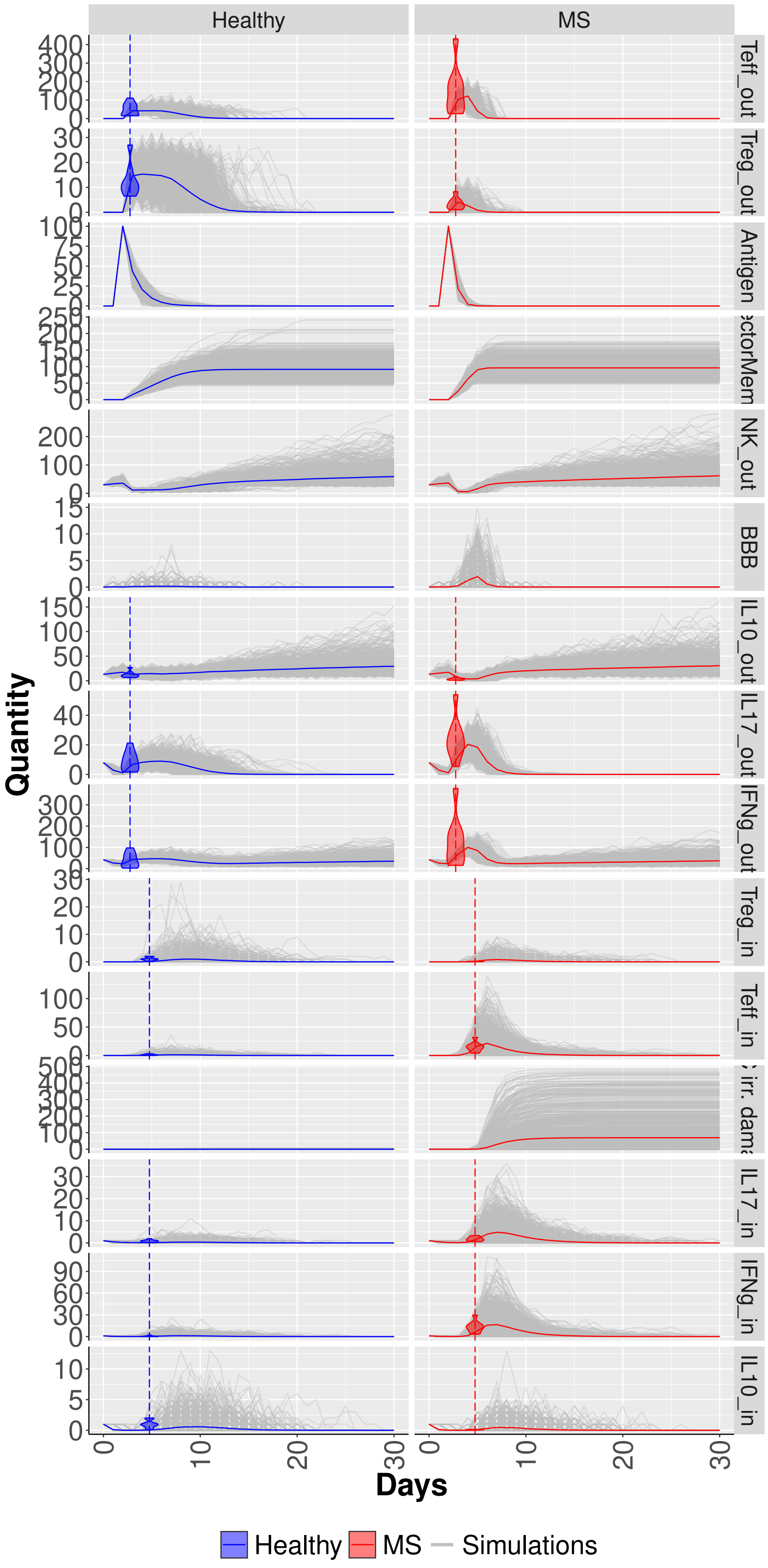

Figure S3 1000 stochastic simulations considering the healthy (first column) and the MS (second column) parameters configuration. The colored bold lines represent the mean traces of the simulations, blue for the healthy and red for the MS scenario. The violin plots are the representation of the real data.

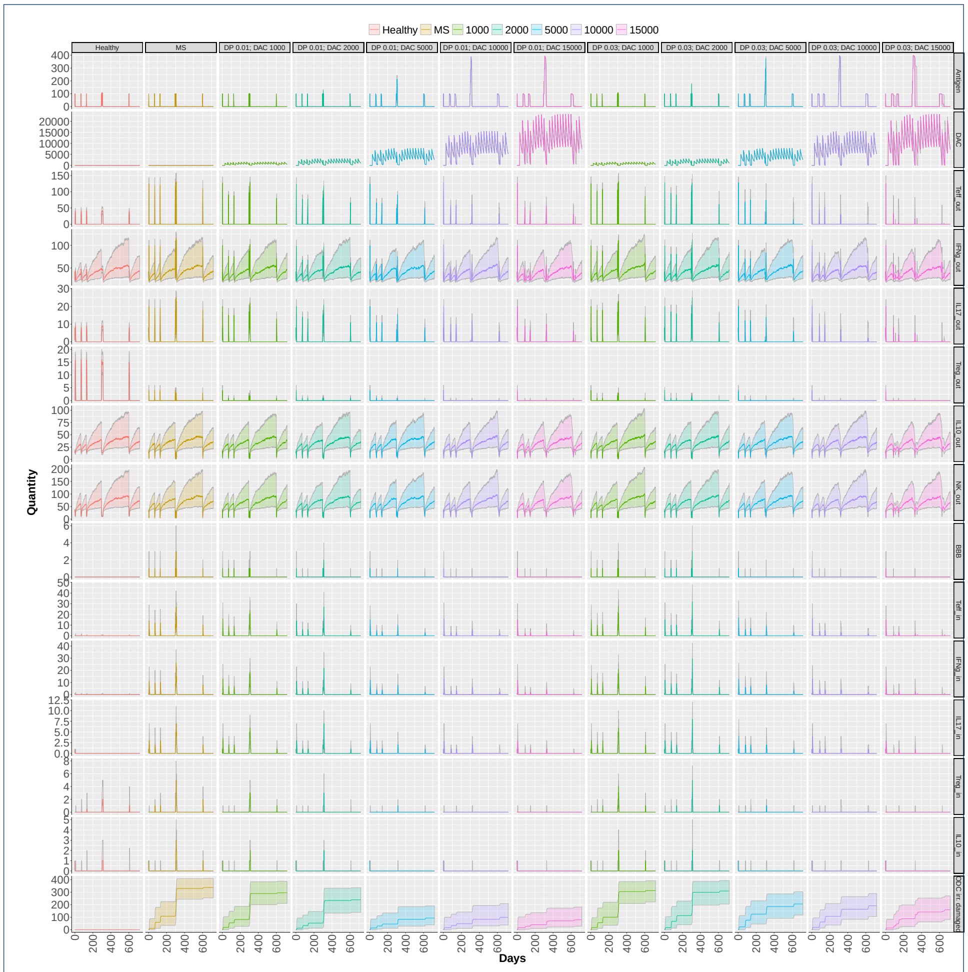

Figure S4 Stochastic simulations considering the **early therapy**. Different colors are associated to quantity of DAC injected for each scenario, from 1000 to 15000 cells. The first two column represent the healthy and MS scenarios. Two drug potencies (called DP) are showed, i.e., 0.01 e 0.03.

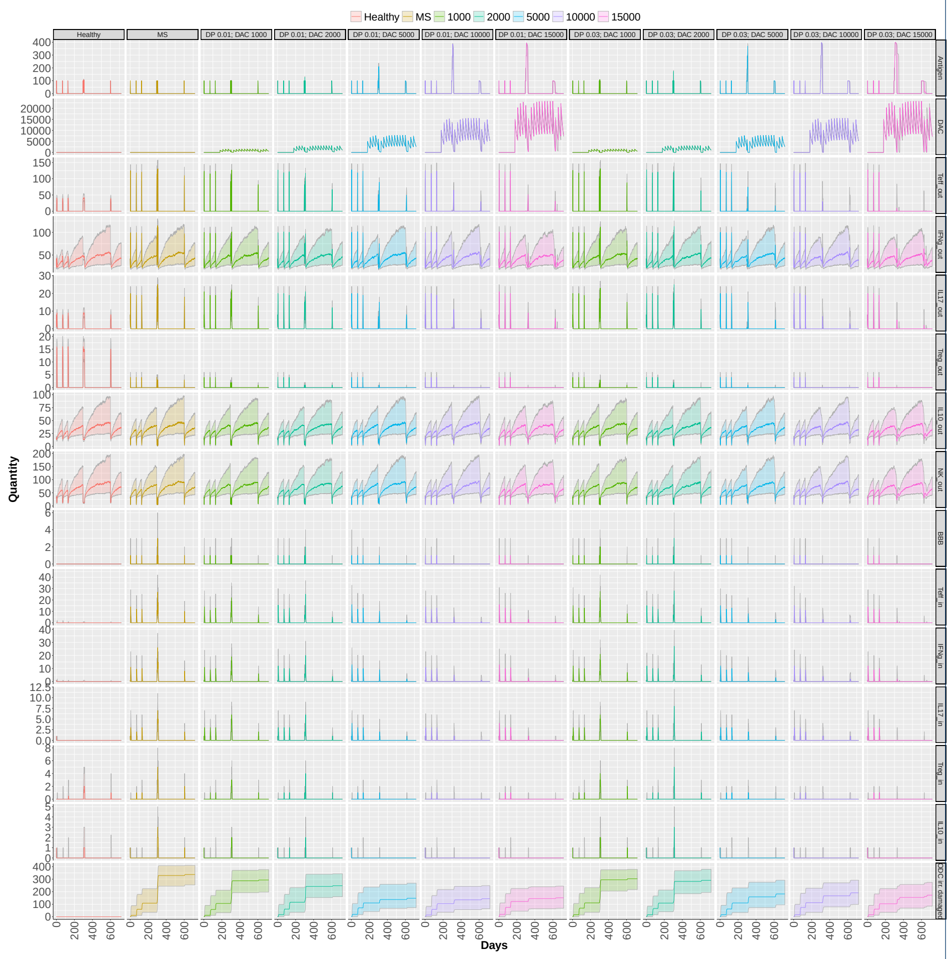

Figure S5 Stochastic simulations considering the **late therapy**. Different colors are associated to quantity of DAC injected for each scenario, from 1000 to 15000 cells. The first two column represent the healthy and MS scenarios. Two drug potencies (called DP) are showed, i.e., 0.01 e 0.03.
